# Association of prior tuberculosis with cardiovascular status in perinatally HIV-1-infected adolescents

**DOI:** 10.1136/openhrt-2024-002960

**Published:** 2024-11-14

**Authors:** Itai M Magodoro, Carlos Eduardo Guerrero-Chalela, Emma Carkeek, Nana Akua Asafu-Agyei, Nomawethu Jele, Lisa J Frigati, Landon Myer, Jennifer Jao, Mpiko Ntsekhe, Katalin A Wilkinson, Robert J Wilkinson, Heather Zar, Ntobeko Ntusi

**Affiliations:** 1 Department of Medicine, University of Cape Town, Observatory 7925, Republic of South Africa; 2 Fundación Cardioinfantil - Instituto de Cardiología, Bogota, Colombia; 3 Department of Pediatrics and Child Health, University of Cape Town, Rondebosch 7700, Western Cape, South Africa; 4 Department of Paediatrics and Child Health, Stellenbosch University, Tygerberg 7505, South Africa; 5 School of Public Health and Family Medicine, University of Cape Town, Observatory 7925, South Africa; 6 Department of Pediatrics, Division of Pediatric Infectious Diseases, Northwestern University Feinberg School of Medicine, Chicago 60611, IL, USA; 7 Department of Internal Medicine, Division of Adult Infectious Diseases, Northwestern University Feinberg School of Medicine, Chicago 60611, IL, USA; 8 Division of Cardiology, University of Cape Town, Observatory 7925, Republic of South Africa; 9 Centre for Infectious Diseases Research in Africa (CIDRI-Africa), Unversity of Cape Town, Observatory 7925, Republic of South Africa; 10 The Francis Crick Institute, London NW1 1 AT, UK; 11 Department of Infectious Diseases, Imperial College, London SW7 2AZ, UK; 12 Extramural Unit of Noncommunicable and Infectious Diseases, South African Medical Research Council , Observatory 7925, Republic of South Africa

**Keywords:** Magnetic Resonance Imaging, EPIDEMIOLOGY, HEART FAILURE, Translational Medical Research

## Abstract

**Background:**

Whether, and how, co-occurring HIV-1 infection (HIV) and tuberculosis (TB) impact cardiovascular status, especially in adolescents with perinatally acquired HIV (APHIV), have not been examined. We hypothesised that APHIV with previous TB disease have worse cardiac efficiency than APHIV without TB, which is mediated by increased inflammation and disordered cardiometabolism.

**Methods:**

APHIV in Cape Town, South Africa, completed 3T cardiovascular magnetic resonance examination and high sensitivity C reactive protein (hsCRP), fasting plasma glucose (FPG), low-density lipoprotein (LDL) and triglyceride measurement. Ventriculoarterial coupling (VAC) was estimated as the ratio of arterial elastance (Ea) to ventricular end-systolic elastance (Ees). Regression models were applied to estimate cross-sectional associations between Ea/Ees ratio and TB status, with decomposition of these associations into direct and mediated effects of hsCRP, FPG and dyslipidaemia, if any, attempted.

**Results:**

We enrolled 43 APHIV with prior TB and 23 without TB of mean (SD) age 15.0 (1.5) and 15.4 (1.7) years, respectively. Prior TB was associated with lower Ea/Ees ratio (0.59 (0.56 to 0.64)) than no TB (0.66 (0.62 to 0.70)), which corresponded to an adjusted mean difference −0.06 (−0.12 to 0.01) (p=0.048). However, previous TB was not associated with increased hsCRP, FPG, LDL or triglycerides nor were hsCRP, FPG, LDL and triglycerides associated with Ea/Ees ruling out their mediated effects in the association between TB and cardiac efficiency.

**Conclusions:**

Previous TB in APHIV is associated with comparatively reduced cardiac efficiency, related to altered VAC. The clinical significance of these findings requires further study, including a wider range of biomarkers of specific immune pathways.

**WHAT IS ALREADY KNOWN ON THIS TOPIC**

- HIV-1 (HIV) in adults is now well-recognised as an independent risk factor for cardiovascular disease (CVD) while growing evidence suggests likely long-term detrimental cardiovascular effects of infection with *Mycobacterium tuberculosis*. Whether and how comorbid perinatal HIV infection (PHIV) and tuberculosis (TB) disease impact cardiovascular status in adolescents is unknown.

**WHAT THIS STUDY ADDS**

- Co-occuring HIV/TB in early life may have synergistic adverse cardiovascular impact.
- It is characterised by comparatively reduced cardiac efficiency, related to altered ventriculoarterial coupling, via mechanisms that remain to be unravelled.

**HOW THIS STUDY MIGHT AFFECT RESEARCH, PRACTICE OR POLICY**

- If corroborated by other studies, research and clinical practice will need to identify effective interventions to optimise the long-term cardiovascular health of young persons with HIV/TB comorbidity.

## Introduction

Chronic or prolonged infections have been linked with heightened risk of cardiovascular disease (CVD).^1^ Chronic infection-induced cellular and humoral immune activation and systemic inflammation may contribute to endothelial dysfunction, hypercoagulability, glucose and lipid metabolism dysregulation, and in turn, atherosclerosis, myocardial inflammation and fibrosis.^2 3^ These pathological changes precede and characterise adverse cardiovascular events in the general population. In this regard, HIV-1 is now well recognised as an independent risk factor for CVD.^4^ Finally, research attention has been drawn to likely long-term detrimental cardiovascular effects of infection with *Mycobacterium tuberculosis (M.tb*).^5 6^ A recent (2020) meta-analysis, summarising extant studies, estimated the risk of incident CVD with prior tuberculosis (TB) to be 1.5 times (risk ratio 1.51 (95% CI 1.16 to 1.97)) higher than without a history of TB.^7^ Of note is that the included studies were largely drawn from high-income countries. If TB does prove to be a risk factor for CVD, the challenge will be immense in sub-Saharan Africa (SSA) where endemic *M.tb* coincides with pandemic HIV, pervasive traditional risk factors like hypertension and cigarette smoking and scant health resources.^8^

However, there is a dearth of studies in SSA examining cardiovascular health in HIV/TB comorbidity, and especially among vulnerable population groups like those with perinatally acquired HIV-1 (PHIV). Children with PHIV are increasingly reaching adolescence and early adulthood due to successful antiretroviral treatment (ART). While many are thriving, a significant proportion faces unprecedented multisystem and multiorgan morbidity, including the cardiovascular system, with prospects for poor long-term outcomes.^9 10^Adolescents with PHIV (APHIV) are exposed to HIV and ART-related cardiotoxicity beginning in infancy, if not *in utero*^11^; and notwithstanding ART, have up to fourfold increased risk of developing TB.^12^ In South Africa, HIV, TB, cerebrovascular disease and CVD are leading causes of premature adult mortality.^13^ Their multimorbidity^14 15^ is very common, raising the possibility that immune activation and systemic inflammation associated with co-occuring HIV/TB likely negatively impacts the cardiovascular system.^5 6^ APHIV experiencing TB may, therefore, be predisposed to premature cardiac morbidity and mortality as they enter adulthood.

Assessment of ventricular arterial coupling (VAC) provides important insights into the pathophysiology of heart failure including its preclinical antecedents.^16^ VAC simultaneously evaluates ventricular performance and arterial haemodynamics, and the degree of matching between the two. Optimal VAC allows the heart to effectively pump blood into the arterial circulation while minimising excess workload.^16^,^18^ Mismatched coupling, where arterial load and ventricular contractility do not correspond, can lead to inefficient cardiac function and potential heart failure. VAC is, therefore, an integrative characterisation of cardiovascular system dynamics.^16^ Combined with assessment of systemic immune changes, evaluation of VAC in APHIV may yield important, actionable insights into early cardiac disease and its pathophysiology. In this cross-sectional study, we aimed to evaluate the association between TB infection and cardiac status among adolescents growing up with PHIV in Cape Town, South Africa. We hypothesised that APHIV with previous TB will have worse VAC than peers without, because of synergism of TB/HIV on immune activation and cardiometabolic dysregulation.

## Methods

We followed the guidelines of the Strengthening the Reporting of Observational Studies in Epidemiology in the conduct and reporting of our analyses.^19^

### Study participants

Participants were drawn from an ongoing longitudinal (parent) study of chronic disease development in Cape Town.^20^ Cohort members include APHIV and their age-matched, sex-matched and community-matched peers without HIV. All APHIV participants were stably on ART, initiated in childhood. For the present analysis, APHIV presenting for a scheduled visit in the parent study were consecutively approached with invitation to undergo cardiovascular magnetic resonance (CMR) examination in addition to routine study procedures. All were eligible for inclusion if they had no current cardiorespiratory symptoms or known structural heart disease, active systemic infection and had no contraindications to CMR.

### Tuberculosis disease

We extracted TB status from electronic medical records. Throughout study follow-up in the parent prospective cohort, participants continued to receive routine care at their primary care sites, including ART and prophylaxis against opportunistic infections. Routine care includes TB screening if symptomatic with a chest radiograph, sputum for GeneXpert MTB/RIF and culture, at the discretion of the primary physician. We defined having previous TB for the present analysis as any history of a clinician-led diagnosis of TB, prescription of standard antituberculosis drug regimen, and/or positive GeneXpert MTB/RIF and/or microscopy and culture of acid-fast bacilli prior to CMR examination. Dates when TB was diagnosed were incomplete to allow us to estimate time intervals between TB episode and CMR examination.

### CMR image acquisition and analysis

The CMR protocol was similar to that previously published by the present authors.^21^ Briefly, we performed all scans at 3 Tesla on a Siemens Skyra MR system (Erlangen, Germany) and acquired long axis (LAX) and short axis (SAX) cines using the breath-hold steady-state free precession sequence. Exams also included T2-weighted imaging for assessment of myocardial oedema and native T1 mapping for diffuse fibrosis. Late-gadolinium enhancement (LGE) images were acquired to assess for scar/fibrosis and postcontrast T1 mapping was performed to calculate extracellular volume (ECV).

Image analysis^22^ was completed offline blinded to PHIV status and using the proprietary CVI42 (Circle Cardiovascular Imaging, Calgary, Canada). Endocardial and epicardial contours were automatically generated with manual correction where warranted. LV end-diastolic volume, LV ejection fraction and left ventricle (LV) mass were calculated on SAX cines. All volumetric and mass data were indexed to height^1.7^ and this is indicated by postscript (i). Strain analysis was done on the SAX and LAX (2Ch, 3Ch and 4Ch) cines using automatic feature tracking. We extracted global peak systolic circumferential and longitudinal strain (GLS), and global peak diastolic circumferential and GLS rates. Motion-corrected T1, T2 and ECV maps were generated using basal, mid-ventricle and apical SAX slices. Global measurements per slice were averaged to yield mean native T1, native T2 and ECV values, while LGE was visually scored for presence (yes/no).

### VAC estimation

The LV and the arterial system are modelled as two coupled elastic chambers. Each chamber is uniquely characterised by elastance, which is the change in pressure for a given change in volume.^16 18^ VAC is assessed by the ratio of arterial elastance (Ea) to ventricular end-systolic elastance (Ees). Ea represents the load on the heart, while Ees signifies load-independent ventricular contractility. Optimal coupling, where energy transfer from the heart to the arteries is maximised, happens when the two elastance are balanced, that is, VAC=1. While cardiac catheterisation with pressure-volume (PV) loop analysis is the gold standard for evaluating VAC, CMR offers a valid non-invasive approximation.^23^,^26^

Ea and Ees are derived from PV loops as:

1. Ea=ESP/SV and
2. Ees=ESP/(ESV – V_0_) or ESP/ESV as V_0_ is assumed to be negligible,

where ESP is end-systolic pressure, ESV is end-systolic volume, SV is stroke volume and V_0_ is the x-axis intercept of the end-systolic pressure–volume relationship (ESPVR). Because mean arterial blood pressure (MAP) closely approximates ESP,^27 28^ both Ea (=MAP/SV) and Ees (= MAP/ESV) can be calculated non-invasively using physically measured MAP (where MAP=([(2× diastolic blood pressure)+systolic blood pressure)/3) and CMR measurements of SV and ESV.^17 29 30^

### HIV markers and antiretroviral treatment history

Participants had their CD4+cell count and HIV viral load (detection limit <40 RNA copies/mL, Roche Cobas AmpliPrep/TaqMan, Pleasanton, California) measured at the time of CMR examination. Data on current and past ART were retrieved from electronic medical records.

### High sensitivity C reactive protein and cardiometabolic markers

The Tina-quant CRPHS immunoturbidimetric assay was used for the quantitative determination of high sensitivity C reactive protein (hs-CRP). Participants were excluded from analysis if hs-CRP>10 mg/dL as this could signify an additional infection or an inflammatory condition.^31^ Fasting plasma glucose (FPG) and lipid subfractions including total cholesterol, triglycerides, high-density lipoprotein and low-density lipoprotein (LDL) were also measured. The last two of three attended brachial blood pressure (BP) measurements taken using electronic sphygmomanometers were averaged, and mean arterial pressure (MAP) determined. Z scores for body mass index (BMI) were calculated from weight (kg) and height (m) and compared with the WHO reference population^32^ to obtain BMI z scores.

### Ethics approval

The Human Research Ethics Committee (HREC) of the Faculty of Health Sciences of the University of Cape Town (HREC 051–2013) approved all study activities as conforming to the ethical guidelines of the 1975 Declaration of Helsinki. Signed informed consent if ≥16 years old or informed parental signed consent and participant assent if <16 years old was obtained prior to participation in the study.

### Patient and public involvement in research

Patients or the public were not involved in the design, or conduct, or reporting or dissemination plans of our research.

### Data availability

Data used in this study are available on reasonable request to the authors.

## Data analysis

**Figure 1:**
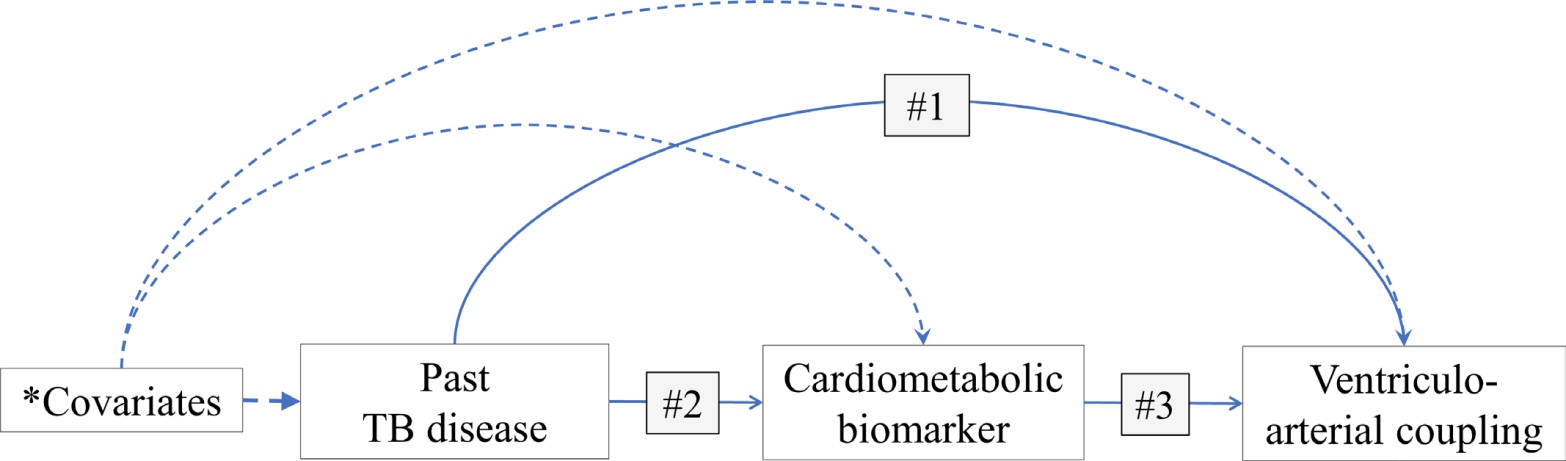
Mediation models of hypothesised indirect effect of inflammation and metabolic dysregulation in the relationship between past tuberculosis disease and ventriculo-arterial coupling in perinatally HIV-1-infected adolescents. BMI, body mass index; NNRTI, non-nucleoside reverse transcriptase inhibitors; PI, protease inhibitor; TB, tuberculosis. *Covariates = age, sex, BMI, HIV viral suppression, and PI and NNRTI exposure.

To test our hypothesis that PHIV with TB is associated with worse VAC than PHIV alone, and that this is driven by increased inflammation/immune activation (measured by hsCRP) and cardiometabolic dysregulation (measured by FPG, LDL and triglycerides), we planned *a priori* a causal mediation analysis using the counterfactual framework. A mediator is defined as a variable that is on the causal pathway between the exposure and outcome of interest (figure 1). Thus, performing mediation analysis allows the identification of pathways by which an exposure impacts an outcome. Formally, mediation is present if the following four conditions are met: (1) the exposure is associated with the outcome of interest; (2) the exposure is associated with the potential mediator; (3) the potential mediator is associated with the outcome of interest and (4) including both exposure and mediator as predictors change the magnitude of association between the outcome of interest and exposure in condition (1).^33^

With past TB infection (yes/no) as the exposure of interest, we built linear regression models of VAC indices to test condition (1), and quintile regression models of, separately, hsCRP, FPG, LDL and triglycerides to test condition (2). To test condition (3), we linearly regressed VAC indices on each of hsCRP, FPG, LDL and triglycerides. All regression models were adjusted for age, sex, BMI, HIV viral suppression, and exposure to protease inhibitors (PI) and non-nucleoside reverse transcriptase inhibitors (NNRTI). However, conditions (2) and (3) were not met (*as outlined in the Results section*), that is, there was no evidence of mediation by any of hsCRP, FPG, LDL and triglycerides and we, therefore, did not proceed to test condition (4). The remainder of the analyses focused on history of TB (exposure) and its association with VAC indices using traditional statistical approaches.

Analyses were conducted on a complete case basis using R, V.3.6.3 (R Foundation for Statistical Computing, Vienna, Austria), and Stata V.17.0 (StataCorp, College Station, Texas). All probability values were two sided, with p values <0.05 considered indicative of statistical significance. Participants’ demographic, HIV and cardiometabolic characteristics were summarised by TB status. Depending on variable scale, we reported values as mean (SD) or median (IQR) or number (%). Differences in cardiac indices and biomarkers according to TB status were examined using linear and quintile regression models, respectively. The β-coefficients from the latter were reported as mean difference in median value. Adjustment was made for age, sex, BMI, HIV viral suppression and exposure to PI and NNRTI.

## Results

### Cohort description

We enrolled 70 APHIV, 43 of whom had previous TB disease and 27 did not (table 1). The two exposure groups had comparable age and sex composition. They also had similar age at ART initiation and total duration of ART exposure. However, their experience with specific ART regimen was different, as were their clinical outcomes. For example, APHIV without TB had longer median (IQR) NNRTI exposure (7.6 (4.7 to 11.0) years)) than their peers with TB (4.2 (0.5 to 8.6) years). Conversely, those with TB had more frequent viral suppression (67%) than APHIV without TB (58%).

**Table 1:** Characteristics of perinatally HIV-1-infected adolescents according to tuberculosis disease status

Characteristic	Clinical tuberculosis disease status	
	**No prior TB**	**Prior TB**
Number	27	43
Age (years, SD)	15.4 (1.7)	15.0 (1.5)
Male sex	16 (59%)	22 (51%)
HIV infection and ART		
Age at ART initiation (years)	3.6 (0.9, 6.7)	3.3 (1.5, 5.5)
Lifetime ART exposure (years)	11.4 (9.3, 13.6)	11.5 (10.1, 13.4)
NRTI exposure (years)	11.4 (9.3, 13.6)	11.5 (10.1, 13.4)
PI exposure (years)	0.6 (0.0, 5.6)	5.9 (0.0, 10.3)
NNRTI exposure (years)	7.6 (4.7, 11.0)	4.2 (0.5, 8.6)
Current CD4+count (cells/mL)	651 (518, 848)	774 (527, 978)
Undetectable HIV viral load	15 (58%)	28 (67%)
Anthropometry		
BMI (kg/m^2^)	20.6 (3.8)	21.3 (5.7)
BMI-for-age z-score	0.00 (1.04)	0.08 (1.49)
Height-for-age z-score	−0.88 (1.25)	−1.03 (1.03)
Weight-for-age z-score	−0.44 (1.22)	−0.37 (1.49)
Blood pressure		
Mean arterial BP (mm Hg)	113.2 (7.5)	111.5 (10.2)
Systolic BP (mm Hg)	104.6 (7.2)	103.3 (9.7)
Diastolic BP (mm Hg)	65.0 (5.1)	64.1 (6.5)

Values are reported as mean (SD) or median (25th, 75th percentile) or number (%).

ARTantiretroviral therpay.BMIbody mass indexBPblood pressureNNRTInon-nucleoside reverse transcriptase inhibitorsPIprotease inhibitorTBtuberculosis

**Figure 2:**
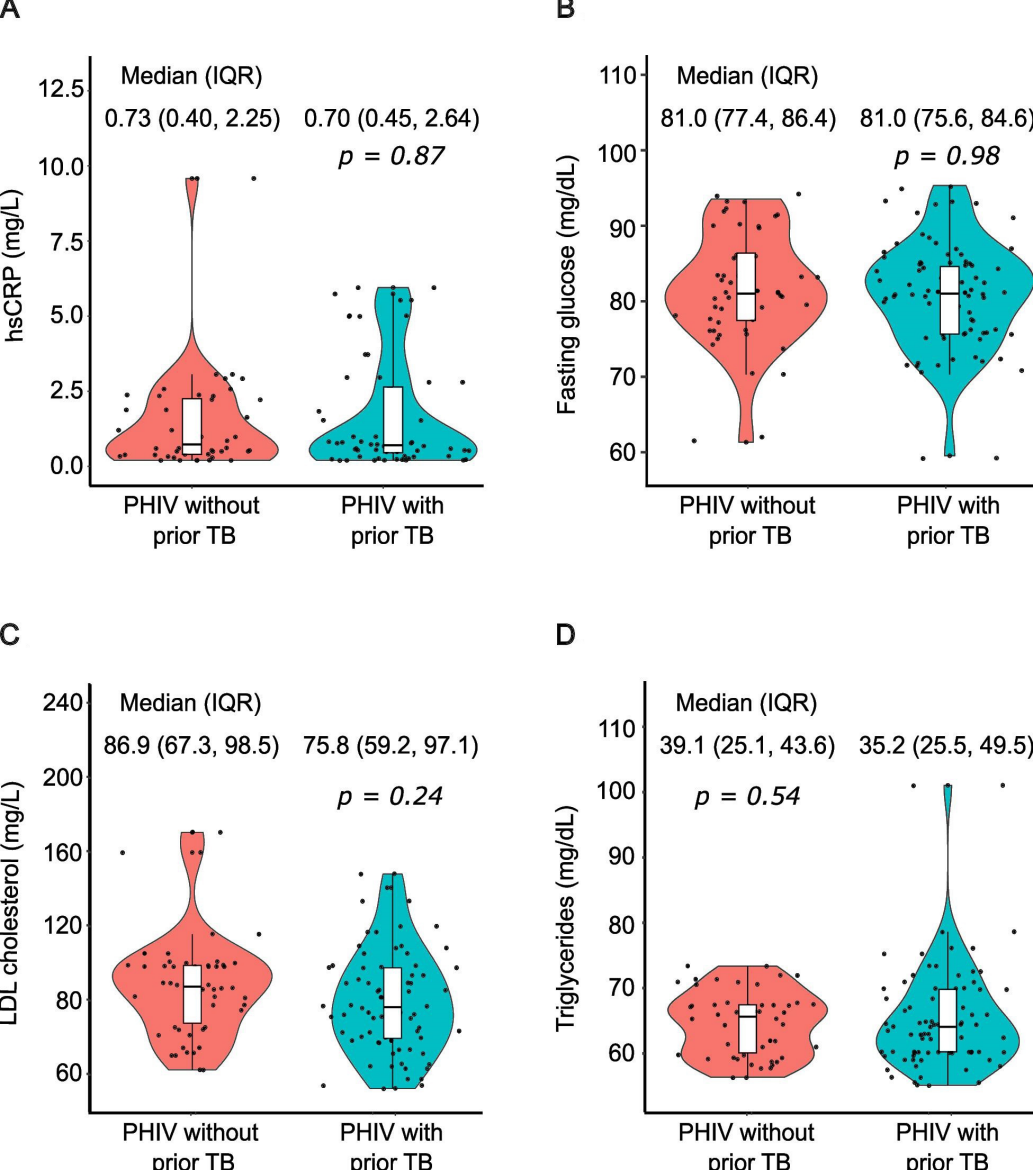
The distribution of cardiometabolic biomarkers among adolescents with perinatally HIV-1 infection according to tuberculosis disease status. LDL, low-density lipoprotein; PHIV, perinatally acquired HIV, TB, tuberculosis.

*hs-CRP and cardiometabolic markers:* there were no statistically significant differences in hsCRP between APHIV with previous TB (median (IQR): 070 (0.45 to 2.64) mg/dL) and their peers without TB (0.73 (0.40 to 2.25) mg/dL; p=0.87) (figure 2A and table 2). When adjusting for potential confounders, having unsuppressed HIV was the only significant predictor of hsCRP: viremia was associated with a 0.53 (0.28 to 1.34) mg/dL (p=0.021) higher median hsCRP compared with undetectable virus. The distribution of FPG, LDL and triglycerides was comparable between the two comparison groups, with and without covariate adjustment (figure 2B–D and table 2).

**Table 2:** Correlates of cardiometabolic biomarkers in perinatally HIV-1-infected adolescents according to tuberculosis disease status

Characteristic	*Adjusted mean difference (95% CI) in median biomarker value							
	hsCRP (mg/L		Fasting glucose (mg/dL)		LDL cholesterol (mg/dL)		Triglycerides (mg/dL)	
	Difference	P value	Difference	P value	Difference	P value	Difference	P value
No prior TB	*Ref*.		*Ref*.		*Ref*.		*Ref*.	
Prior TB	0.10 (−1.28, 1.48)	0.88	−0.4 (−4.9, 4.1)	0.86	−3.8 (−23.6, 15.9)	0.70	−4.2 (−18.1, 9.67)	0.55
Male	*Ref*.		*Ref*.					
Female	−0.49 (−1.41, 0.42)	0.29	−4.2 (−8.8, 0.4)	0.071	3.5 (−18.5, 25.6)	0.76	8.2 (−4.0, 20.2)	0.19
1-year age increase	0.29 (−0.10, 0.68)	0.15	−1.9 (−3.5,–0.5)	0.011	−4.8 (−10.0, 0.44)	0.073	0.1 (−4.4, 4.5)	0.98
2.5 kg/m^2^ increase in BMI	0.11 (−0.35, 0.57)	0.65	0.7 (−0.3, 1.7)	0.18	1.6 (−2.9, 6.0)	0.49	0.1 (−4.9, 5.0)	0.11
PI exposure (*vs none*)	−0.01 (−1.15, 1.13)	0.98	−3.8 (−9.2, 1.6)	0.11	8.8 (−16.5, 35.0)	0.61	9.1 (−2.1, 20.2)	0.78
NNRTI exposure (*vs none*)	0.47 (−0.66, 1.60)	0.41	2.0 (−5.7, 9.8)	0.62	−7.3 (−45.0, 3.3)	0.66	−2.4 (−17.6, 12.8)	0.78
Undetectable HIV viral load	*Ref*.		*Ref*.		*Ref*.		*Ref*.	
Viremia	0.53 (0.28, 1.34)	0.021	−0.6 (−5.6, 4.3)	0.80	−20.9 (−45.1, 3.2)	0.090	−11.3 (−22.7, 0.1)	0.052

Values are mean (lower, upper bound 95% CI CI).

* β- coefficients (95% CI CI) from quantile regression models reported as mean difference in 50th percentile (or median) value.

† Model adjusted for tuberculosis (TB) history, sex, age, body mass index (BMI), protease inhibitor (PI) and non-nucleoside reverse transcriptase inhibitor (NNRTI) exposure, and HIV viremia.

hsCRPhigh sensitivity C reactive protein

### Prior TB and left ventricular indices

The differences in cardiac indices by, and their association with TB are summarised in tables3  4. We found no evidence of significant differences in ventricular mass, volumes and diastolic function according to TB infection. Similarly, there were no significant differences by TB status in scarring (LGE presence: 52.0 vs 48.4%; p=0.79), diffuse fibrosis (ECV (%): 28.5 vs 29.1%; p=0.51) or myocardial tissue inflammation (T2 (ms): 38.2 vs 38.3 ms; p=0.69). Systolic function measured by LVEF was similar between APHIV with previous TB (mean (95% CI) 63.5 (61.2 to 64.6)%) and those without TB (60.6 (58.8 to 62.4)%; p=0.048). This was the case too when systolic function was measured by peak systolic strain. For example, mean (95% CI) peak GLS was −20.7 (−21.6 to 19.7)% for APHIV with prior TB and −20.4 (−21.3 to 19.5)% (p=0.72) for those without prior TB (table 3).

**Table 3:** Left ventricular parameters in perinatally HIV-1-infected adolescents according to tuberculosis disease status

Parameter	Clinical tuberculosis disease status		
	No prior TB	Prior TB	P value
Number	27	43	
Ventricular-arterial coupling			
Ea (mm Hg/mL)	1.50 (1.39, 1.62)	1.49 (1.39, 1.60)	0.93
Height-adjusted Ea (mm Hg/mL.m^2.7^)	0.45 (0.39, 0.51)	0.46 (0.42, 0.50)	0.65
Ees (mm Hg/mL)	2.34 (2.15, 2.53)	2.59 (2.38, 2.80)	0.039
Height-adjusted Ees (mm Hg/mL.m^2.7^)	0.70 (0.61, 0.78)	0.80 (0.71, 0.89)	0.033
Ea/Ees ratio	0.66 (0.62, 0.70)	0.56 (0.53, 0.64)	0.015
Ventricular wall strain and function			
Peak circumferential strain			
Systolic strain (%)	−21.5 (2.3)	−21.3 (2.7)	0.77
Diastolic strain rate (/s)	1.76 (1.62, 1.90)	1.77 (1.64, 1.90)	0.90
Peak longitudinal strain			
Systolic strain (%)	−20.4 (-21.3,–19.5)	−20.7 (-21.6,–19.7)	0.72
Diastolic strain rate (/s)	1.61 (1.47, 1.74)	1.63 (1.55, 1.71)	0.83
LVEF (%)	60.6 (58.8, 62.4)	63.5 (61.2, 64.6)	0.048
LV geometry			
LVMi (g/m^2.7^)	56.8 (53.6, 59.9)	56.2 (53.4, 57.5)	0.78
LVEDVi (mL/m^2.7^)	37.2 (34.8, 39.1)	36.6 (35.0, 38.9)	0.71
LVESVi (mL/m^2.7^)	14.9 (13.5, 15.9)	13.4 (12.7, 14.9)	0.088
Myocardial tissue			
Presence of LGE	52.0 (32.7, 70.8)	48.4 (31.4, 65.8)	0.79
Extracellular volume (%)	28.5 (27.5, 29.5)	29.1 (28.3, 29.9)	0.51
Native T2 (ms)	38.2 (37.5, 38.9)	38.3 (37.4, 39.2)	0.69

Values are reported as mean (95% CI CI).

Postscript (i)=indexed to (height).2.7.

Ea, arterial elastance; Ees, end-systolic ventricular elastanceLGElate-gadolinium enhancementLVleft ventricleLVEDVLV end-diastolic volumeLVESVLV end-systolic volumeLVMLV massTBtuberculosis

**Table 4:** Association between ventriculoarterial coupling and prior clinical tuberculosis disease in perinatally HIV-1-infected adolescents according to tuberculosis disease status

Parameter	Mean difference in height-adjusted* ventriculoarterial coupling indices					
	Ea		Ees		Ea/Ees ratio	
	β (95% CI)	P value	β (95% CI)	P value	β (95% CI)	P value
No prior TB	Ref.		Ref.		Ref.	
Prior TB	0.01 (−0.07 to 0.08)	0.84	0.11 (0.01 to 0.22)	0.031	−0.09 (−0.15 to 0.01)	0.048
Female	Ref.		Ref.		Ref.	
Male	0.09 (0.02 to 0.16)	0.010	0.27 (0.11 to 0.42)	0.001	−0.07 (−0.13 to 0.01)	0.037
1-year age increase	−0.02 (−0.05 to 0.010)	0.038	−0.6 (−0.11 to 0.020	0.006	0.01 (−0.01 to 0.04)	0.29
2.5 kg/m^2^ increase in BMI	−0.01 (−0.02 to 0.01)	0.43	0.0 (−0.03 to 0.03)	0.90	−0.01 (−0.03 to 0.01)	0.37
PI exposure (vs none)	−0.01 (−0.12 to 0.09)	0.82	0.04 (−0.10 to 0.18)	0.58	−0.04 (−0.10 to 0.02)	0.21
NNRTI exposure (vs none)	0.05 (−0.03 to 0.13)	0.24	0.0 (−0.18 to 0.18)	0.98	0.01 (−0.10 to 0.11)	0.90
Undetectable HIV viral load	Ref.		Ref.		Ref.	
Viremia	−0.01 (−0.09 to 0.07)	0.84	0.12 (−0.02 to 0.26)	0.092	−0.08 (−0.03 to 0.18)	0.51

* Indexed to (height).2.7. Model adjusted for TB history, sex, age, body mass index (BMI), protease (PI) and non-nucleoside reverse transcriptase inhibitor (NNRTI) exposure, and HIV viremia.

Ea, arterial elastance; Eesend-systolic ventricular elastanceNNRTInon-nucleoside reverse transcriptase inhibitorsPIprotease inhibitors

### Ventricular arterial coupling

Regarding our primary endpoint, both adjusted and unadjusted analyses found a lower VAC ratio in PHIV with prior TB than PHIV alone. Prior TB was associated with an adjusted mean difference in Ea/Ees ratio of −0.09 (−0.16 to 0.01) (p=0.048) relative to no prior TB. This difference was driven by the higher Ees in PHIV with TB than PHIV without TB (adjusted mean difference: 0.11 (0.01 to 0.22) mm Hg/mL.m^2.7^; p=0.031) given the similar Ea (0.01 (−0.07 to 0.08) mm Hg/mL.m^2.7^; p=0.84). The other significant determinant of Ea/Ees ratio was sex whereby men had lower Ea/Ees ratio than their female counterparts (adjusted mean difference: −0.09 (−0.15 to 0.01); p=0.048).

## Discussion

Our study aimed to assess the impact of TB/HIV comorbidity on CMR-assessed cardiac status in perinatally APHIV in South Africa. We found that prior TB versus none was associated with comparatively worse cardiac efficiency related to mismatched arterial elastance and ventricular end-systolic elastance. This association was not accounted for by mediated effects of increased hsCRP, FPG, LDL triglycerides, as measures of systemic inflammation and cardiometabolism. Neither did we find significant TB-related differences in ventricular volumes, dimensions and function. The clinical significance of the observed altered VAC associated with TB requires further studies, as do the underlying immunological mechanisms.

VAC influences cardiac stroke work and cardiac efficiency. Stroke work quantifies the energy expended to generate stroke volume during each cardiac cycle. It is determined by the stroke volume (LVSV) and the MAP. An increase in stroke work (LVSV×MAP) indicates a greater mechanical load on the heart.^16 17^ Cardiac efficiency, on the other hand, is the ratio of stroke work to the total energy expended (stroke work/myocardial oxygen consumption). It measures cardiac effectiveness in converting energy into useful mechanical work.^16 17 30^ Thus mismatched Ea and Ees can result in increased stroke work and decreased cardiac efficiency. In contrast, when coupling is well matched, stroke work is optimised while cardiac efficiency is increased. Experimental data^17^ suggest that stroke work is maximised with a VAC ratio equals 1 while mechanical and energy efficiency are maximised at a VAC ratio equals 0.5. Nevertheless, in extensive studies involving healthy adult populations, the VAC ratio typically ranged from 0.6 to 0.8.^34 35^ This observation implies that under normal physiological conditions, the parameters are configured to optimise mechanical and energy efficiency. Indeed, both hypertensive and heart failure patients have been found to have reduced Ea/Ees (<0.6) compared with healthy controls.^35 36^

We are not aware of any published data on VAC, Ea and Ees in adolescent HIV infection in SSA. A recent European guideline (2019) proposed normal adult values for Ea and Ees of 2.2 mm Hg/mL and 2.3 mm Hg/mL, respectively.^16^ We are also not aware of equivalent guidelines for children and adolescents. Individual reports from North America and Western Europe have reported, for example, Ea of 1.6 mm Hg/mL and Ees of 0.9 mm Hg/mL in healthy adolescents^30^ and Ea of 2.1 mm Hg/mL and Ees of 3.3 mm Hg/mL in obese adolescents,^37^ whereas we found an Ea of 1.5 mm Hg/mL and Ees of 2.6 mm Hg/mL for adolescents with HIV/TB coinfection. One possible interpretation of our results is that HIV/TB coinfection in adolescents may be associated with increased Ees and non-elevated Ea. APHIV with prior TB had lower VAC ratio than APHIV without TB. This would suggest maximisation of mechanical and energy efficiency as opposed to stroke work.^17^ However, the VAC ratio with TB/HIV comorbidity (Ea/Ees=0.56) may represent cardiovascular efficiency below the optimal range.^36 38^ Low values of the VAC ratio imply inappropriately high ventricular end-systolic elastance for a particular level of arterial elastance. In our study, arterial elastance was equal between the two TB groups whereas ventricular end-systolic elastance was higher among those with TB than their counterparts without. There are at least two mechanisms by which a low VAC ratio might portend adverse cardiovascular outcomes. It has been shown in adults that a high Ees increases the cardiac energy cost of increasing stroke volume,^36 38^ and that low VAC ratio is associated with increased diastolic stiffness and diastolic dysfunction.^36^

Our findings corroborate the evidence from the few available studies showing an association between TB/HIV comorbidity and heart failure or its antecedents. Bakari *et al* found that a history of TB was associated with a threefold higher likelihood of LV systolic heart failure (adjusted OR: 3.01 (1.32 to 11.56)) among adult persons living with HIV (PLWH) in Tanzania,^39^ whereas Ndongala *et al* in Lesotho reported a sixfold higher likelihood of heart failure (adjusted OR: 6.25 (1.24 to 31.48)) with prior TB.^40^ In the latter, their definition of heart failure included pulmonary heart disease, that is, right heart failure in the presence of pulmonary hypertension. Similarly, past TB was independently predictive of subclinical cardiopulmonary dysfunction (adjusted OR: 2.3 (1.2 to 4.4)) in South African adolescents with PHIV.^41^ This is the only study to date, to our knowledge, focusing on APHIV. Noteworthy, this study defined cardiopulmonary dysfunction as any of RV systolic dysfunction, LV diastolic or systolic dysfunction or abnormal mean pulmonary arterial pressure, in conjunction with abnormal spirometry or a deficient 6 min walking test. Detracting from these studies, including ours, is their small sample size, cross-sectional design, convenient sampling, and hospital-facility or health-facility-based enrolment.

The differences by TB status in systemic inflammation or cardiometabolic markers were not statistically significant, and thus precluded mediation causal analyses. Our study relied solely on hsCRP—versus multiple pathway markers—as a measure of inflammatory activity. It remains an important and urgent priority, from mechanistic and intervention points of view, to delineate the role of immune activation and systemic inflammation in TB/HIV-associated cardiovascular changes. Relatedly, assessing Ea and Ees over traditional indices like LVEF has the advantage of improved discrimination of changes in ventricular performance, arterial load or both.^16^ Formally evaluating the utility of this approach to risk identification and stratification in APHIV will be an important extension of our work. However, this presupposes that our observed and otherwise subclinical findings of mismatched arterial elastance and ventricular end-systolic elastance have prognostic significance.

## Strengths and limitations

We believe that our study is among the first to examine the relationship between HIV/TB comorbidity and VAC in APHIV. This is an understudied but potentially at-high-risk population subgroup for premature cardiovascular ill-health. We employed CMR which has superior reproducibility, higher accuracy and sensitivity for cardiac assessment compared with other imaging modalities. Furthermore, CMR-based assessment of VAC parameters has been demonstrated to provide estimates that are comparable to those derived from invasively measured intracardiac pressure–volume loops.^42^

VAC can detect subtle changes in cardiovascular function at earlier stages compared with traditional indices like LVEF, which may mask heart dysfunction until it is well advanced.^43^ However, we lacked detailed TB infection history. Neither did we comprehensively assess immune activation and systemic inflammatory pathways. These shortcomings precluded granular mechanistic insights. Besides, our study was cross-sectional precluding causal interpretations and may have been underpowered to detect differences in biomarkers, and thus to undertake a causal mediation analysis. Equally cautionary was our definition of TB, which included clinical diagnoses and thus probably misclassification biases. Notwithstanding, our findings do shed new light on a subject of growing clinical and public health concern. These findings remain to be replicated, and their long-term significance mapped out. Future work should include HIV uninfected adolescents as controls to better assess and understand the impact of TB/HIV comorbidity on cardiovascular health.

## Conclusion

We demonstrate that previous TB in APHIV is associated with comparatively reduced cardiac efficiency, related to mismatched arterial elastance and ventricular end-systolic elastance. The clinical significance of these findings requires further studies, including a wider range of biomarkers of specific immune pathways. However, it is clear that greater efforts are needed for enhanced and effective interventions to optimise the management of APHIV particularly as they enter adulthood and become increasingly independent of their caregivers.

## Data Availability

Data are available upon reasonable request.
